# New York Academy of Medicine

**Published:** 1880-06

**Authors:** 


### NEW YORK ACADEMY OF MEDICINE.

---

Dr. J. C. Peters delivered a memorial address upon the late Dr. Freeman J. Bumstead. At the close of the eulogy, a portrait, in crayon, of Dr. Bumstead was presented to the academy.

OBLIQUE SECTION OF SKIN IN SURGICAL OPERATIONS.

Dr. John H. Packard read a paper entitled, "On some important advantages to be secured by oblique section of the skin in surgical operations." The paper being open for discussion,

Dr. James R. Wood said that the late Dr. Valentine Mott had advocated, especially in operations on the scalp and face, oblique incisions. To stop the hemorrhage, he did not use torsion, but he left his wounds open until hemorrhage ceased. He then closed the wound, if on the scalp, by twisting the hair and tying it, so as not to make punctured wounds with the needle. In this way he would get union by the first intention. Dr. Mott used to teach that this beveling of the integument was very important, especially in operation on the face of females. We can now, however, make this method more uniformly applicable, because we have now water and carbolized catgut with which to staunch hemorrhage. Dr. Wood advocated the method suggested by Dr. Packard, although he, as a rule, believed in the open treatment of wounds. Dr. Wood had used catgut, but he had not always been able to prevent suppuration. He was satisfied that there was a good deal in the catgut ligature, but it was not perfectly reliable. Dr. Wood said that he once witnessed a beautiful operation in Philadelphia, many years ago, in which Dr. Pancoast, Sr., made a new nose. He beveled off the integument of the forehead; then grooved the edges of the nasal opening where the new nose was to be. The two parts were then fitted into each other, and the union was rapid and perfect.

Dr. Frank P. Hamilton said that he could see one objection to Dr. Packard's plan, viz: that by making oblique incisions where the skin was very thin there might sometimes be sloughing. On the other hand, better apposition might be secured, and a larger part of the incision would be in the more vascular part of the tissues. These were two very apparent advantages.

Dr. Alfred C. Post said that he had not practised this method, but he had long been satisfied that broad surfaces should be applied to each other in closing wounds. He had been in the habit of bringing such surfaces together, using deep sutures and keeping the parts in place by splints. In this way he had secured union in fistulæ, etc., where the edges alone would have healed together with difficulty.

In operations about the vagina the method of bringing broad serfaces together, as suggested by Dr. Post, was absolutely necessary.

Dr. Lente said that he thought the suggestion of Dr. Packard would be a very useful one. In reference to closing up wounds at once, he thought it preferable not to do this in country practice, where the patients could only be seen once in two or three days. It was safer, he thought, to carry out the open treatment, and thus lessen the possibility of accidents.

Dr. A. C. Post said that in cases where a large cavity is left after the operation there is no question about the necessity of keeping the wound open; but there was often an error in keeping the drainage tube in too long. If not removed in two or three days after the operation, it was likely to become only a source of irritation.

Dr. Webster said that some six or eight weeks ago he witnessed the removal of a deeply seated orbital tumor. The incision was made vertically, the tumor enucleated, and the wound closed with silk sutures. The wound closed immediately, there was a very slight scar, and the result seemed to be quite as good as that in the case reported by Dr. Packard.

Dr. Robert F. Weir had twice used Dr. Packard's method, and thought favorably of it. As regards the possibility of sloughing, he said the incisions in corneæ for contaract had for years been made obliquely, yet there had been no sloughing in such cases, so far as he was aware, although the tissue was a non-vascular one.

Dr. F. P. Hamilton said that Mr. Pierson, in his directions for opening cold abscesses, stated that the incisions should be made obliquely. He had followed this direction a few times with satisfactory results.

Dr. Harwood related a case of removal of tumor of the face. The wound was closed with sutures and dressed with carbolized oil. The next day the union had taken place, and a week later the scar was very slight. In this case the incision was vertical, and the dressing applied in the ordinary way.

Dr. Packard, in concluding the discussion, said that, 1st, as regards Dr. Wood's open method, he was entirely in accord with him. He regarded the open, fresh air a very much maligned element. But it did not seem to him that the idea of the open method militated against his theory of oblique incision at all. There were classes of cases for which each was most applicable. The remarks of Dr. Wood reminded him of what Dr. Paget says of the distinction between immediate union and union by first intention. He thought the distinction an important one. In immediate union, there is union with no effusion of lymph. Now, by the oblique method, this union is more likely to be obtained. And the point is, in this incision, to so cut the skin as to have the edges closely in apposition, and the sides of the cavity so brought together as to prevent the formation of any cavity; the atmospheric pressure helps to secure this result.

2d—In reference to the danger of sloughing, he referred to cases of skin-grafting. The facts regarding this were a sufficient answer to the suggested danger.

3d—in regard to how much bevelling of the skin there should be, a case was necessary to show it perfectly. The incision must be made very obliquely. It should be made at an angle of 90° or more. There is all the difference in the world between such extreme obliquity and a slight amount. Some surgeons had totally misapprehended him on this point.

4th—Dr. Packard was aware that the idea of oblique incisions was not new. He recollected Dr. Pancoast's operation for rhinoplasty, referred to by Dr. Wood. This was, however, for a special operation only. The points he (Dr. Packard) urged, were the uniform advantages of the oblique incision in securing accurate apposition of the skin, and in preventing the formation of a cavity.

5th—In reference to whether it should include the subcutaneous tissue, he did not mean it to include this, except, perhaps, in tumors of the breast. The use of hot water, as a hæmostatic, he considered one of the greatest additions to modern surgery.

